# Supplementary material for: The multi-dimensional challenges of controlling respiratory virus transmission in indoor spaces: Insights from the linkage of a microscopic pedestrian simulation and SARS-CoV-2 transmission model
Source: PLoS Comput Biol. 2024 Mar 28;20(3):e1011956. doi: 10.1371/journal.pcbi.1011956 (PMC11003685; doi:10.1371/journal.pcbi.1011956)
Supplement: Attachment — Submitted filename: MS_SSO_PLOSCOMBIO_R1_Responses_to_Reviewers.docx [file pcbi.1011956.s002.docx]

**Responses to the Reviewers’ Comments – R1**

**PLOS Computational Biology**

**The multi-dimensional challenges of controlling** **respiratory virus transmission in indoor spaces:**

**Insights from the linkage of a microscopic pedestrian simulation** **and SARS-CoV-2 transmission model**

**AU:** In response to the insightful feedback provided by the reviewers, we have revised the original manuscript. All changes have been highlighted in the manuscript. We have worked on the reviewers’ comments in detail and responded to all comments point by point in this document. In addition to those, we have revised two figures in the manuscript: Figure 10 to enhance visual clarity, and Figure S1 to align with latest version of our simulator tool, SamenSlimOpen App.

**Reviewer #1:** The paper describes a thorough mechanistic modelling study of COVID transmission in a restaurant setting. The work uses carefully considered and integrated sub-models to represent activity, movement, viral exposure and risk. The authors should be congratulated on an important and comprehensive study. I believe it adds an important element to the body of work on viral transmission and mitigation and I support its publication.

**AU:** The authors thank the reviewer for their constructive and supportive comments.

**R1:** There are a number of issues that should be addressed before publication and these are listed below.

Major points:

* The model includes three routes of transmission, including fomite. However, as the authors say, the baseline scenario does not favour this route as there are no shared surfaces in the scenario. This is made clear in the interpretation. However, for the main scenario the modelling of surface touch is a little hard to assess. If I have understood correctly, the tables and chairs around them are the surfaces that can be contaminated and shared, effectively acting as a transmission route. That seems reasonable but doesn't seem to cover all of the potential for cross contamination. Many studies have focused on high touch surfaces such as door handles that have the potential to pass contamination to multiple users of the space. This might be appropriate in this scenario for the entrance/exit as well as for the bathrooms (and the other surfaces therein). Also, are the coat rack and pay register included as touchable surfaces? It would be good to clarify this and to discuss it in the limitations section.

**AU:** The comment regarding the shared surfaces as a potential route for transmission indicates an important point for many infectious diseases, especially those whose transmission through surfaces play a major role, such as Ebola or chicken pox. However, for COVID-19, evidence shows that the virus mainly spreads through respiration (Greenhalgh et al., 2021; Miller et al., 2021; Zhang et al., 2020), and transmission through surfaces is limited (Mondelli et al. 2020; Meyerowitz et al. 2021; Lewis et al., 2021; Zhang et al., 2021; Cheng et al., 2022). Experimental studies have shown that SARS-CoV-2 can be transmitted through the environment (Gerhards et al., 2023) but this transmission is related to a build-up of virus resulting from long resident times in a shared environment (i.e., the cumulative deposition of droplets over time), rather than through high touch surfaces. Consequently, for the case study in this particular manuscript, authors decided to consider the surface-mediated transmission only through the main activity areas of the customers, which are their tables and chairs. The model is however capable of incorporating high touch surfaces. The main motivation for the model to include the virus transmission mechanism through (high touch) surfaces is that our model aims to be generic in representing the transmission routes. Following the suggestion of the reviewer, we added the following explanation about the contribution of surfaces to the Discussion section (pages 11 and 12, lines 576-589):

**“In many infectious diseases, especially those whose transmission through surfaces plays a major role such as Ebola or chicken pox, shared surfaces can be an important infection transmission route. PeDViS can simulate the virus transmission mechanism through surfaces along with the aerosols and droplets and is thereby generic in representing all transmission routes relevant to respiratory pathogens. However, for COVID-19, evidence shows that the virus mainly spreads through respiration (Greenhalgh et al., 2021; Miller et al., 2021; Zhang et al., 2020), and transmission through surfaces is limited (Mondelli et al. 2020; Meyerowitz, Richterman, Gandhi, et al. 2021; Lewis et al., 2021; Zhang et al., 2021; Cheng et al., 2022). Experimental studies in cats have shown that SARS-CoV-2 can be transmitted through the environment (Gerhards et al., 2023) but this transmission is primarily associated with the accumulation of the virus in the environment over prolonged time in a shared space rather than being linked to high-touch surfaces. Therefore, for this particular case study, this low probability transmission route is considered only through the main activity areas of the customers, which are their tables and chairs.** **However, other scenarios with conditions more favourable to fomite transmission, including the possible transmission through other high-touch surfaces such as door handles, coat rack or pay register, can be examined to better understand the potential for contribution by this route, for SARS-CoV-2 and other pathogens.”**

- Cheng, P., Luo, K., Xiao, S., Yang, H., Hang, J., Ou, C., Cowling, B.J., Yen, H.L., Hui, D.S., Hu, S. and Li, Y., 2022. Predominant airborne transmission and insignificant fomite transmission of SARS-CoV-2 in a two-bus COVID-19 outbreak originating from the same pre-symptomatic index case. Journal of hazardous materials, 425, p.128051.

- Gerhards, N.M., Gonzales, J.L., Vreman, S., Ravesloot, L., van den Brand, J.M., Doekes, H.P., Egberink, H.F., Stegeman, A., Oreshkova, N., van der Poel, W.H. and de Jong, M.C., 2023. Efficient direct and limited environmental transmission of SARS-CoV-2 lineage B. 1.22 in domestic cats. Microbiology Spectrum, 11(3), pp.e02553-22.

- Greenhalgh, T., Jimenez, J.L., Prather, K.A., Tufekci, Z., Fisman, D. and Schooley, R., 2021. Ten scientific reasons in support of airborne transmission of SARS-CoV-2. The lancet, 397(10285), pp.1603-1605.

- Lewis, D., 2021. COVID-19 rarely spreads through surfaces. So why are we still deep cleaning. Nature, 590(7844), pp.26-28.

- Meyerowitz, E.A., Richterman, A., Gandhi, R.T. and Sax, P.E., 2021. Transmission of SARS-CoV-2: a review of viral, host, and environmental factors. Annals of internal medicine, 174(1), pp.69-79.

- Miller, S.L., Nazaroff, W.W., Jimenez, J.L., Boerstra, A., Buonanno, G., Dancer, S.J., Kurnitski, J., Marr, L.C., Morawska, L. and Noakes, C., 2021. Transmission of SARS‐CoV‐2 by inhalation of respiratory aerosol in the Skagit Valley Chorale superspreading event. Indoor air, 31(2), pp.314-323.

- Mondelli, M.U., Colaneri, M., Seminari, E.M., Baldanti, F. and Bruno, R., 2021. Low risk of SARS-CoV-2 transmission by fomites in real-life conditions. The Lancet Infectious Diseases, 21(5), p.e112.

- Zhang, N., Chen, X., Jia, W., Jin, T., Xiao, S., Chen, W., Hang, J., Ou, C., Lei, H., Qian, H. and Su, B., 2021. Evidence for lack of transmission by close contact and surface touch in a restaurant outbreak of COVID-19. Journal of Infection, 83(2), pp.207-216.

- Zhang, R., Li, Y., Zhang, A.L., Wang, Y. and Molina, M.J., 2020. Identifying airborne transmission as the dominant route for the spread of COVID-19. Proceedings of the National Academy of Sciences, 117(26), pp.14857-14863.

**R1:** * Although there is an exploration of the uncertainty in the relationship between viral exposure and risk of infection that includes different levels of susceptibility, the assumption of average infectivity is not explored particularly. Studies suggest that the viral load varies over many orders of magnitude. This is likely to have a strong effect on risk and is best thought of as a continuous spectrum rather than normal and superspreader. It would be good to acknowledge this as a central source of uncertainty to be borne in mind when interpreting these results.

**AU:** S6 Fig shows the model expectations of how the number of infections might vary depending on the emission rate, which here is assumed to be directly proportional to an individual’s viral load. We have added text to the main manuscript (page 12, lines 597-606) to reflect the suggestions of the reviewer:

**“While many of the model parameters may affect the absolute virus exposure, predicted infection risks were found to be robust to changes in most of the parameters explored (see details in S6 Fig-S12 Fig). Infection risks are most sensitive to different levels of emission rates. Here, these are assumed proportional to individual viral loads, which are known to be highly heterogeneous, both between individuals as over the course of the infection (Chen et al., 2021). Heterogeneity in infectiousness may, among other factors, be an important source of heterogeneity in observed outbreak sizes. In a sensitivity analysis on the emission rates, the average number of secondary infections varied from 0.09 to 5.40 (S6 Fig), reflecting that, while most individuals will on average not contribute to onward transmission, some may affect many (Adam et al., 2020). PedViS can be used to further disentangle the sources of heterogeneity that together result in the highly overdispersed outbreak sizes observed for this pathogen.”**

and (page 12, lines 625-628):

**“In QVEmod, the assignment of infectious agents is randomised as well as whether a specific virus exposure results in infection. Further, sources of individual-level heterogeneity such as in infectiousness and respiratory activities can be examined to better understand the drivers behind superspreading events.”**

- Adam, D.C., Wu, P., Wong, J.Y., Lau, E.H., Tsang, T.K., Cauchemez, S., Leung, G.M. and Cowling, B.J., 2020. Clustering and superspreading potential of SARS-CoV-2 infections in Hong Kong. Nature Medicine, 26(11), pp.1714-1719.

- Chen, P.Z., Bobrovitz, N., Premji, Z., Koopmans, M., Fisman, D.N. and Gu, F.X., 2021. Heterogeneity in transmissibility and shedding SARS-CoV-2 via droplets and aerosols. Elife, 10, p.e65774.

**R1:** * p24, eqn(23) - why does the amount of virus on the hand not diminish through transfer to mucous membranes? On a related note, the epsilon parameter in Table 3 is given with units of per hour but has no such units in the supplementary information. This value does seem very low. The SI doesn't fully explain how it is derived. Could this be included for transparency.

**AU:** The authors thank reviewer for their remarks. The model uses the estimated transferred amount from hands to mucous membranes on the face as the indicator of the surface-mediated exposure (pages 19 and 20, lines 976-981). The reduction of the particles on hand through transfer is assumed to be small enough that it can be covered within the inactivation rate on the skin (Supporting Information, page 12, lines 576-580). This assumption has now been explicitly added to the Supporting Information, page 11, lines 558-560:

*“***The reduction of particles on the hand that results from transfer to facial membranes is not considered as a separate process, but rather assumed small enough to be covered within the inactivation rate on the skin in the calculation of** ε**.”**

As detailed in Supporting Information, page 11 lines 553-558, ε represents an aggregated value for virus transfer rate from hands to facial membranes and has four components. The unit of ε is indeed units per hour, and it is derived by these four components as below:

fractional virus transfer rate from hands to facial membranes (1/hr)

= touching surface ratio between the hands and facial membranes (cm^2^/cm^2^)

* frequency of touching the face (touch/hr)

* virus transfer efficiency from hands to face (1/touch)

* exp (- inactivation rate (1/hr) * time (hr)) (unitless proportion, since exponential decay indicates the decrease at a rate proportional to its current value)

For the initial derivation of the value, the highest and lowest values found in the literature has been used:

ε_H_ = 0.01*23*0.339*exp(-0.8) = 0.035

ε_L_ = 0.01*15.7*0.2*exp(-88) ≈ 0

Since it was calculated to be between 0%-3.5%, the baseline value is selected as 1%, and a sensitivity analysis was conducted for values between 0.1%-10%, which provided robust results (S12 Fig), i.e., 10-fold decrease in the transfer rate resulted in 2% decrease, and 10-fold increase resulted in 18% increase in the expected number of infections.

The explanations about the unit and the derivation of ε have been added to Supporting Information, page 12 lines 582-587:

**“In this context, ε can be calculated as follows:**

**ε (1/hr) = touching surface ratio between the hands and facial membranes (cm^2^/cm^2^) * frequency of touching the face (touch/hr) * virus transfer efficiency from hands to face (1/touch) ***

**exp (-inactivation rate (1/hr) * time (hr))**

**“The calculation using the above-described estimates results in a fractional transfer rate of 0%-3.5%. Given the substantial uncertainty around this metric, we performed a sensitivity analysis over a wider range.”**

**R1:** Minor points:

**R1:** p3, l114 - Wells-Riley (and elsewhere)

**AU:** Edited as suggested.

**R1:** p3, l146 - '... across the range of possible...'?

**AU:** Edited as suggested.

**R1:** p9, l357 - '... for an extended period of time...'

**AU:** Edited as suggested.

**R1:** p14, l498 - '...less efficiently...'

**AU:** Edited as suggested.

**R1:** p16, l606 - 'Nomad' and 'NOMAD' used interchangably in numerous places

**AU:** All *“Nomad”* phrases have been updated to *“NOMAD”*.

**R1:** p16, l617 - sentence doesn't make sense - too many words or words missing?

**AU:** The sentence has been corrected and now reads “**Further, sources of individual-level heterogeneity, such as in infectiousness and respiratory activities, can be examined towards a better understanding of the drivers of superspreading events.**”

**R1:** p26, Table 3 - What does 'Proportion of pathogen exerced to hands' mean? Is that a typo?

**AU:** The typo has been corrected as “**Proportion of pathogen excreted to hands**”.

**R1:** p12, fig 8 - the y-axis should have correct labels (Infection risk?) and the existing ones moved perhaps horizontally away from the axes to avoid confusion.

**AU:** Figure 8 has been updated as suggested by the reviewer. Y-axis has been updated as “Exposure load”, while the scenarios of each run have been moved to the top of each plot horizontally. The figure title has been updated as “The impact of face masks and better ventilation on virus exposure in the case study”.

**Fig 8. The impact of face masks and better ventilation on virus exposure in the case study.**

**R1:** p27, Table 3 - The breathing rates look low for adults. I would expect around 600 L hr-1. There are tabulated values widely used for environmental assessment rather than calculating from tidal breathing and rate of breathing.

**AU:** The authors thank the reviewer for their remark. We base our estimates on the assumption that a typical adult exchanges approximately 0.4 L or 0.5 L (for females and males respectively) and do so at a rate of 12 times per minute (Pleil et al., 2021; Hallett et al., 2020). The inhalation rate can be calculated by multiplying these two, which resulted in about 4.8 L for females and 6 L for males per minute. These parameters are considered robust default values (Pleil et al., 2021). Individual variations do exist due to size, age, and gender. In our manuscript, we use 4.8*60 = 228 L as our base case of hourly inhalation rate, which increases up to 432 L per hour, depending on the respiratory activity considered (Bernardi et al. 2017).

- Bernardi, N.F., Snow, S., Peretz, I., Orozco Perez, H.D., Sabet-Kassouf, N. and Lehmann, A., 2017. Cardiorespiratory optimization during improvised singing and toning. Scientific reports, 7(1), p.8113.

- Hallett, S., Toro, F. and Ashurst, J.V., 2018. Physiology, tidal volume. In StatPearls. Treasure Island (FL): StatPearls Publishing. <https://www.ncbi.nlm.nih.gov/books/NBK482502/>.

- Pleil, J.D., Wallace, M.A.G., Davis, M.D. and Matty, C.M., 2021. The physics of human breathing: Flow, timing, volume, and pressure parameters for normal, on-demand, and ventilator respiration. Journal of breath research, 15(4), p.042002.

**Reviewer #2:** I commend the authors for a detailed and well-crafted manuscript. The PeDVis model developed by the authors is an effective way to limit the transmission of respiratory viruses in future pandemics. A crucial aspect of pandemic management plans is the implementation of interventions aimed at reducing transmission within indoor spaces. However, I have a few suggestions for minor corrections that could be made in the manuscript.

**AU:** The authors thank the reviewer for their constructive and supportive comments.

**R2:** Lines 70-71 state that the duration, closeness, and number of contacts the infectious individual has while visiting the indoor space drive transmission. This statement is accurate, but it would benefit from being supported by a reference.

**AU:** The authors thank the reviewer for their remark. Two studies, Ferretti et al. (2023) and Wilson et al. (2022), have been added as references to support the corresponding sentence (page 2, line 71).

- Ferretti, L., Wymant, C., Petrie, J., Tsallis, D., Kendall, M., Ledda, A., Di Lauro, F., Fowler, A., Di Francia, A., Panovska-Griffiths, J. and Abeler-Dörner, L., 2023. Digital measurement of SARS-CoV-2 transmission risk from 7 million contacts. Nature, pp.1-3.

- Wilson, A.M., Aviles, N., Petrie, J.I., Beamer, P.I., Szabo, Z., Xie, M., McIllece, J., Chen, Y., Son, Y.J., Halai, S. and White, T., 2022. Quantifying SARS‐CoV‐2 infection risk within the Google/Apple Exposure Notification framework to inform quarantine recommendations. Risk Analysis, 42(1), pp.162-176.

**R2:** Authors should cite multiple studies when using the phrase "These studies show" in line 73 (lines 71-75).

**AU:** The authors thank the reviewer for their remark. Two additional studies, Hoeben et al. (2021) and Pouw et al. (2020), have been incorporated into the sentence accordingly. The sentence is now read as follows (page 2, lines 73-76):

**“These studies show, amongst other things, that the changes in the interaction patterns as a result of COVID-19 pandemic are very context dependent (Hoeben et al., 2021; Pouw et al. 2020), and that habitual interaction patterns are difficult to change (van Schaik et al. 2022).”**

- Hoeben, E.M., Bernasco, W., Suonperä Liebst, L., Van Baak, C. and Rosenkrantz Lindegaard, M., 2021. Social distancing compliance: A video observational analysis. PloS one, 16(3), p.e0248221.

- Pouw, C.A., Toschi, F., van Schadewijk, F. and Corbetta, A., 2020. Monitoring physical distancing for crowd management: Real-time trajectory and group analysis. PloS one, 15(10), p.e0240963.

**R2:** In line 228, I suggest that "breath" should be rewritten as "breathe" to make the sentence clearer and grammatically correct.

**AU:** The typo has been corrected as suggested by the reviewer.
