# Supplementary material for: The multi-dimensional challenges of controlling respiratory virus transmission in indoor spaces: Insights from the linkage of a microscopic pedestrian simulation and SARS-CoV-2 transmission model
Source: PLoS Comput Biol. 2024 Mar 28;20(3):e1011956. doi: 10.1371/journal.pcbi.1011956 (PMC11003685; doi:10.1371/journal.pcbi.1011956)
Supplement: Attachment — Submitted filename: MS_SSO_PLOSCOMBIO_R2_Responses_to_Reviewers.docx [file pcbi.1011956.s003.docx]

**Responses to the Reviewers’ Comments – R2**

**PLOS Computational Biology**

**The multi-dimensional challenges of controlling** **respiratory virus transmission in indoor spaces:**

**Insights from the linkage of a microscopic pedestrian simulation** **and SARS-CoV-2 transmission model**

**AU:** In response to the feedback provided by the reviewers and the editor, we have addressed the suggestions and revised the manuscript. We have also revised the details of 4 references (2 in the main Manuscript file, 2 in the Supporting Information document) that were published as preprints during the initial submission but are now available as journal articles.

**Reviewer #1:** Thank you to the authors for addressing the comments so carefully and thoroughly.

**AU:** The authors thank the reviewer for acknowledging the revised manuscript.

**R1:** I was happy with all of the comments with the exception of the response below where I had one further suggestion.

R1 - comment 1 response. The addition to the text is helpful but is slightly circular - it seems to say COVID-19 mainly is spread via other routes so we haven't dealt with shared surfaces properly. I'm oversimplifying and I think the final sentence is helpful pointing to these other surfaces. I would ask though that the second and third sentences are revised. Other studies have shown that high touch surfaces can be important for transmission. For example, Miller et al. (2022), have shown with modelling of a shared space that touch of only briefly contaminated surfaces can be important when taken over large numbers of people. Experimental measurement of the variability of surface contamination in real environments is difficult and I believe the current wording is too definitive. I would suggest the following amendment or similar:

"However, for COVID-19, evidence shows that the virus mainly spreads through respiration (Greenhalgh et al., 2021; Miller et al., 2021; Zhang et al., 2020), and transmission through surfaces *may be* limited (Mondelli et al. 2020; Meyerowitz, Richterman, Gandhi, et al. 2021; Lewis et al., 2021; Zhang et al., 2021; Cheng et al., 2022). Experimental studies in cats have shown that SARS-CoV-

2 can be transmitted through the environment (Gerhards et al., 2023) but this transmission is primarily associated with the accumulation of the virus in the environment over prolonged time in a shared space rather than being linked to high-touch surfaces, although Miller et al. (2022) show that high-touch surfaces can be important in crowded environments."

Miller, Daniel, et al. "Modeling the factors that influence exposure to SARS‐CoV‐2 on a subway train carriage." Indoor Air 32.2 (2022): e12976.

**AU:** The authors thank the reviewer for their suggestion. The corresponding text in the Discussion section (lines 586-595) has been updated exactly as follows:

“However, for COVID-19, evidence shows that the virus mainly spreads through respiration (Greenhalgh et al., 2021; Miller et al., 2021; Zhang et al., 2020), and transmission through surfaces may be limited (Mondelli et al. 2020; Meyerowitz, Richterman, Gandhi, et al. 2021; Lewis et al., 2021; Zhang et al., 2021; Cheng et al., 2022). It can, however, not be ruled out and might play a role in specific scenarios. Experimental studies in cats, for example, have shown that SARS-CoV-2 can be transmitted through the environment (Gerhards et al., 2023). This transmission is primarily associated with the accumulation of the virus in the environment over prolonged time in a shared space rather than being linked to high-touch surfaces. Alternatively, simulation studies (Miller et al., 2022) illustrate that high-touch surfaces could potentially play a role in crowded settings such as train carriages, where some surfaces are potentially shared by many different individuals.”

Miller et al. (2022) has been added to References.

**R1:** In addition, on scanning the paper I noticed that two of the mechanisms in Fig 11 had the same number: 6. Inhalation and 6. Contaminate Surfaces.

**AU:** The authors thank to reviewer for their attentive review. The correct numbering of the process is “**5. Inhalation**”, and Figure 11 has been corrected as follows:

**Reviewer #2:** I would like to commend the authors for their diligent efforts in significantly improving the manuscript.

**AU:** The authors thank the reviewer for acknowledging the revised manuscript.
